# Preparation of purified perikaryal and synaptosomal mitochondrial fractions from relatively small hypothalamic brain samples

**DOI:** 10.1016/j.mex.2016.05.004

**Published:** 2016-05-19

**Authors:** David S. Kiss, Istvan Toth, Gergely Jocsak, Agnes Sterczer, Tibor Bartha, Laszlo V. Frenyo, Attila Zsarnovszky

**Affiliations:** a Department of Physiology and Biochemistry, Szent Istvan University Faculty of Veterinary Sciences, Budapest, Hungary; b Department and Clinic of Internal Medicine, Szent Istvan University Faculty of Veterinary Sciences, Budapest, Hungary; c Division of Comparative Medicine, Yale University School of Medicine, New Haven, CT, USA; d Department of Animal Physiology and Animal Health, Szent Istvan University Faculty of Agricultural and Environmental Sciences, Godollo, Hungary

**Keywords:** Isolation of mitochondrial fraction for oxygen-consumption measurements, Percoll, Gradient fractionation, Respiration measurement, Clark-type electrode

## Abstract

In order to measure the activity of neuronal mitochondria, a representative proof of neuronal processes, physiologically relevant mitochondrial samples need to be gained as simply as possible. Existing methods are, however, either for tissue samples of large size and/or homogenous microstructures only, or are not tested for mitochondrial function measurements. In the present article we describe a gradient fractionation method to isolate viable and well-coupled mitochondria from relatively heterogeneous histological microstructures such as the hypothalamus. With this new method, we are able to isolate a sufficient amount of functional mitochondria for determination of respiratory activity, in a short period of time, using affordable equipment.

• Verified by electron microscopy, our method separates highly enriched and well-preserved perikaryal and synaptosomal mitochondria. Both fractions contain minimal cell debris and no myelin. Respiratory measurements (carried out by Clark-type electrode) confirmed undisturbed mitochondrial function providing well-evaluable records. The demonstrated protocol yields highly viable mitochondrial subfractions within 3 h from small brain areas for high-precision examinations. Using this procedure, brain regions with relatively heterogeneous histological microstructure (hypothalamus) can also be efficiently sampled.
• Up to our present knowledge, our method is the shortest available procedure with the lowest sample size to gain debris-free, fully-viable mitochondria.

## Introduction

In the last decades, oxygen consumption measurements of neuronal mitochondria has been put into focus as a representative proof of neuronal activity and/or impairment (reviewed by Kann and Kovacs [1]). Compared to other, conventional methods (e.g. PCR, Western Blot, immunohistochemistry), oxygen consumption measurements allow the scientists to gather radically distinct information of the underlying neuronal processes and cellular metabolism. Besides the obvious advantages, a major drawback of oxygen consumption measurements is that, for reliable results, fully functional and viable mitochondria need to be obtained.

In the past, numerous isolation techniques have been developed in order to gain well-purified mitochondrial fractions suitable for neuronal activity analyses [2], [3], [4], [5], [6], [7]. Initial approaches were based on differential fractionation using either continuous (self-generated) or discontinuous sucrose gradient [8], [9], [10], [11]. By these methods, mitochondria were exposed to osmotic conditions that significantly scaled down mitochondrial viability. To tackle the problem, later, the osmotically active sucrose was changed to a Ficoll gradient. This change seemed to be very promising as it improved almost all important parameters (yield, effort, etc.) [12], [13], [14], [15]. Although Ficoll allowed isolation of well-purified synaptic and extrasynaptic mitochondrial fractions with acceptable viability (i.e. respiratory properties), the procedure required a considerably long ultracentrifugation step [16], [17]. Ultracentrifugation inevitably needs an equipment of higher grade and more work load, on the other hand, it causes damages in mitochondrial membranes as well as it does not remove all subcellular elements that could seriously mask the native respiratory activity and functional integrity of the separated mitochondria [18], [19]. To get around these drawbacks, Percoll was introduced into the field of mitochondrial fractionation. This product has held out with its extraordinary properties over Ficoll offering the possibility of rapid, but delicate fractionation together with a precise separation of subcellular components [20], [21]. Methods using Percoll gradient are available for some time now, and they seem very reliable for obtaining viable mitochondria that are well-suitable for respiration measurements.

Originally, respiration measurements were developed for hepatocytes [22], therefore the sample size and a possible biochemical contamination were less limiting in terms of measurement accuracy. Later, when this promising method was modified for brain tissues, scientists encountered a serious problem: from brain samples, obtaining a critically large amount of functional mitochondria was unexpectedly difficult (due to differences in mitochondrial functions among brain regions; intrinsic heterogeneity; obstacles in sampling because of size, shape, consistency, and tissue vulnerability). As a solution, researchers increased the sample size that, especially in case of smaller brain areas, often resulted in grouped samples of several animals [23], [24]. By some experimental conditions, working with pooled mitochondrial samples originated from several individual specimens provides acceptable data [33], however there is an evident and always growing effort to be able to examine bioenergetics of small and well-defined brain regions of different individual specimens [34], [35]. Most studies on mitochondrial functions usually process such target brain areas that are not only large enough, but also have favorable histological properties regarding the preservation of the mitochondrial viability during the isolation procedure [25], [26], [27], [28], [29], [30], [31], [32].

In this article, we describe a detailed method to isolate sufficient amount of functional mitochondria from relatively small brain samples for further determination of their respiratory activity. During the development, our goal was to radically refine the existing methods [19], [20], [21] in order to (1) measure small brain parts (such as 5 mg of a rat hypothalamus) (2) in a short period of time (3) using relatively simple and affordable equipment. The hypothalamus is not only a small brain region but also contains relatively large amount of white matter (such as *chiasma opticum*, *fornix* and ventral parts of internal capsule, etc.) and parts of *pia mater.* This “contamination” is hardly avoidable during an ordinary dissection, and the biochemical compounds (such as phospholipids, long chain fatty acids or molecules with steroid scaffold) impair the reliability of the mitochondrial respiration measurement by uncoupling the respiration chain or disrupting the membrane integrity of mitochondria [36], [37].

The isolation procedure was tested (quality testing) by electron microscopy for high resolution morphological confirmation and mitochondrial respiration measurements (carried out by Clark-type oxygen electrode) for viability verification. Quality testing proved that the presented method is suitable for mitochondrial respiration measurement of hypothalamic samples (and probably other brain regions of similarly heterogeneous microstructure) in various experimental conditions, furthermore, it also separates the synaptosomal and somal (i.e. extrasynaptosomal) mitochondrial subfractions.

## Materials and methods

In this protocol, we describe the steps of preparing synaptosomal and extrasynaptosomal mitochondrial fractions from rat hypothalami. For quality testing of this method 10–15 week-old Wistar rats were used. We followed the guidelines laid down by the National Institutes of Health, the use of animals was approved by the University Committee on Animal Use at Szent Istvan University Faculty of Veterinary Sciences, Hungary (Permit Number: XIV-I-001/2202-4/2012).

### Presented protocol

A detailed step-by-step protocol including the necessary reagents and preparation of equipment can be found in Appendix A and B of Supplementary materials. Note that some of the solutions should be freshly prepared and some of them can be stored up to one week before experimental processing. All solutions and equipment have to be perchilled and kept at 4 °C throughout the whole procedure.

The presented method consists of three major steps: (1) preparation of a “crude mitochondrial” fraction; (2) purification of the crude mitochondrial fraction with a simplified Percoll gradient; (3) clearing off the Percoll from the purified sample.

#### Preparation of crude mitochondrial fraction from brain tissue

After decapitation, the brain region of interest is quickly extracted and immediately placed into 750 μl ice-cold isolation buffer (isolation buffer always contains EGTA, unless indicated otherwise). The dissecting steps are performed on 4 °C (cold room; tools kept on ice: brain mold, blades, scissors, spatulas, etc.). Before the separation procedure, all samples should be homogenized separately using a perchilled teflon-on-glass tissue homogenizer with a motorized pestle (600–800 rpm; ten strokes of each samples). The homogenizer is always cleaned between two samples with fresh isolation buffer. As a result of the homogenization, undamaged somal mitochondria and synaptosomes are released, beside other subcellular organelles and cell debris.

**Fig. 1:**
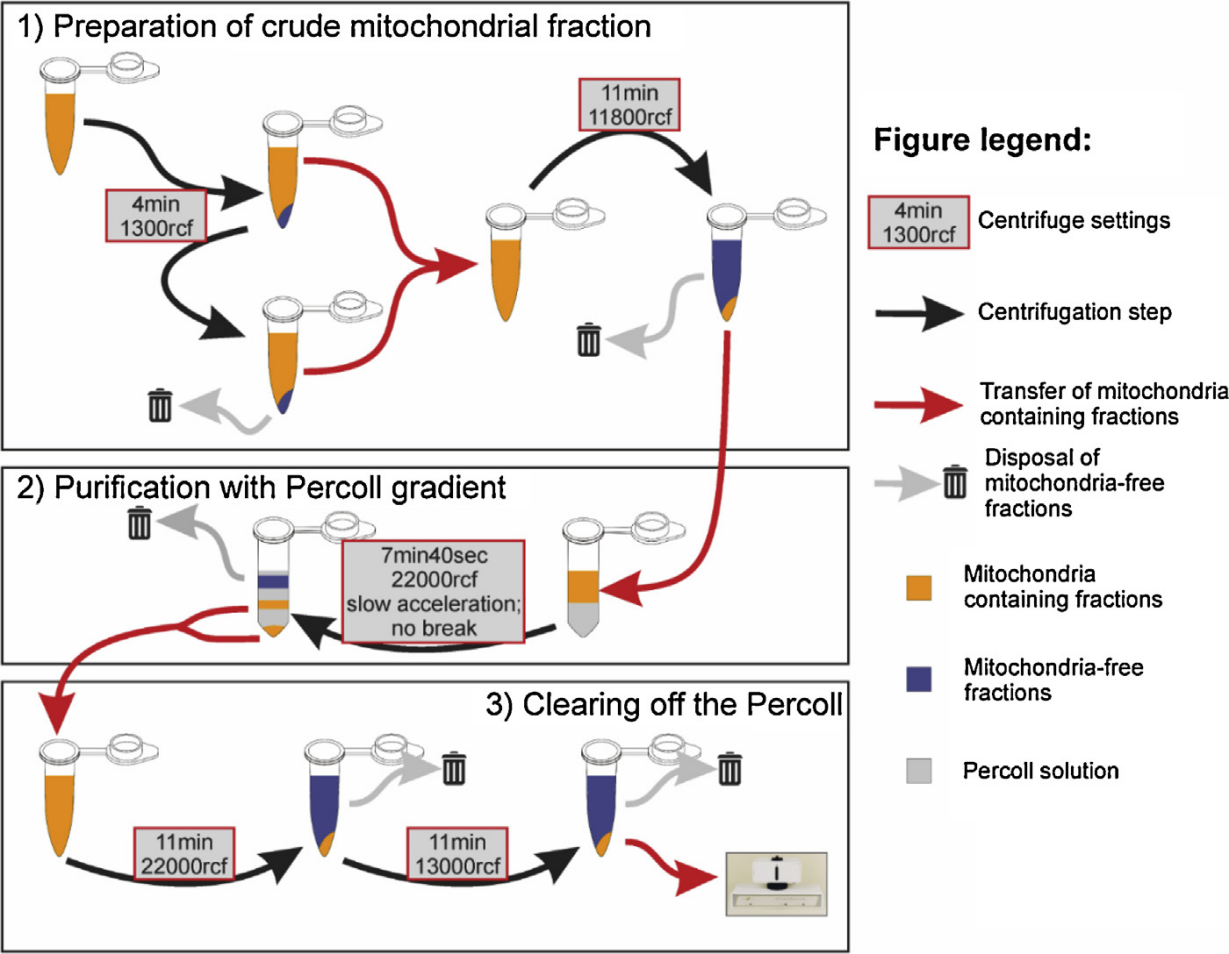
Centrifugation steps of mitochondrial fractionation as described in this method. In the first phase (1) crude mitochondrial fraction is gained as a result of the classical differential fractionation, followed by Percoll gradient fractionation (2) in order to remove un-useful cell organelles, cell debris, and finally (3), Percoll is removed from the samples before biochemical and physiological assays.

After homogenization, all buffer that contains mitochondria and cell debris has to be recollected from the homogenizer and put into a microcentrifuge tube (1.5 ml Eppendorf tube). Then the so-called “crude mitochondrial fraction” is obtained by the following centrifugation steps (all steps are at 4 °C; use fixed angle rotor bench-top centrifuge). For easier understanding, all the steps (together with gradient fractionation on Percoll) are visualized on Fig. 1; and also outlined in Appendix A of Supplementary materials.

As a first step, each homogenized sample is spun at 1300 rcf (3700 rpm) for 4 min. After centrifugation, the supernatant is collected in an empty Eppendorf tube, while the pellet is resuspended in 750 μl isolation buffer, then it is spun again with the same settings (first step repeated to release mitochondria from large cell debris). After the second spin the supernatant is pooled together with the former supernatant collected from the first step. At this point, the pellet can be discarded since it contains no more useful elements. In the next step, the two supernatants collected in one tube are spun together at 13000rcf (11800 rpm) for 11 min. During the centrifugation, the mitochondria sink into the pellet, thus the floating, debris containing supernatant can be poured off and discarded. The pellet is resuspended in 500 μl of isolation buffer and called the “crude mitochondrial fraction”, which still contains many contaminating particles (other cell organelles, myelin, cell debris, etc.) and should be further purified for better physiological measurement.

#### Purification with a Percoll gradient fractionation

**Fig. 2:**
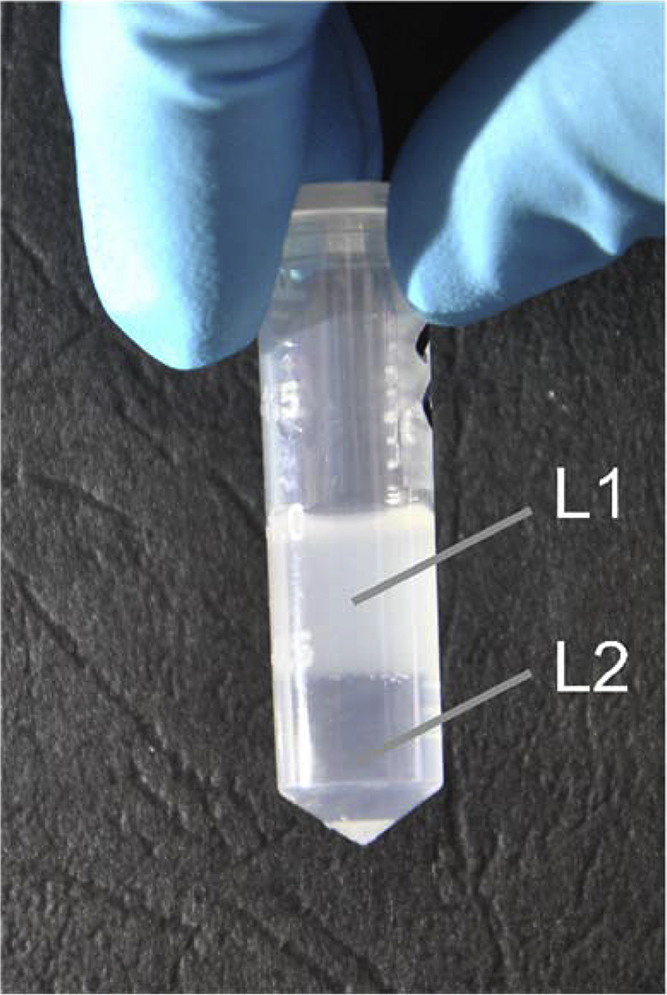
Preparation of two-layer Percoll gradient in 2 ml conical microcentrifuge tube. The 0% Percoll layer containing the opalescent sample (L1) is overlaid onto the 15% Percoll solution (L2) using an extremely low flow rate. To do so, the pipette tip should be first conducted along the tube wall to build a narrow fluid bridge that helps the suspension (L1) of sample to flow gently on top of the L2 layer.

For further purification, a simplified discontinuous Percoll gradient is used that merely consists of a 15% and a 0% Percoll layer (filtered Percoll stock solution is diluted to 15% with isolation buffer, see Appendix A of Supplementary materials). This separates the mitochondria and synaptosomes from other, non-useful elements; on the other hand, easy enough to prepare even in a small-sized Eppendorf tube. First 500 μl of 15% Percoll (layer L2) is be put in a special “Percoll tube” (2 ml, of conical shape; see Fig. 2). The homogenized sample containing the crude mitochondrial fraction (as a matter of fact this is the 0% Percoll layer, L1) is carefully layered on top of the 15% Percoll by a fine pipette avoiding even the slightest mixing of the two layers (Fig. 2).

**Fig. 3:**
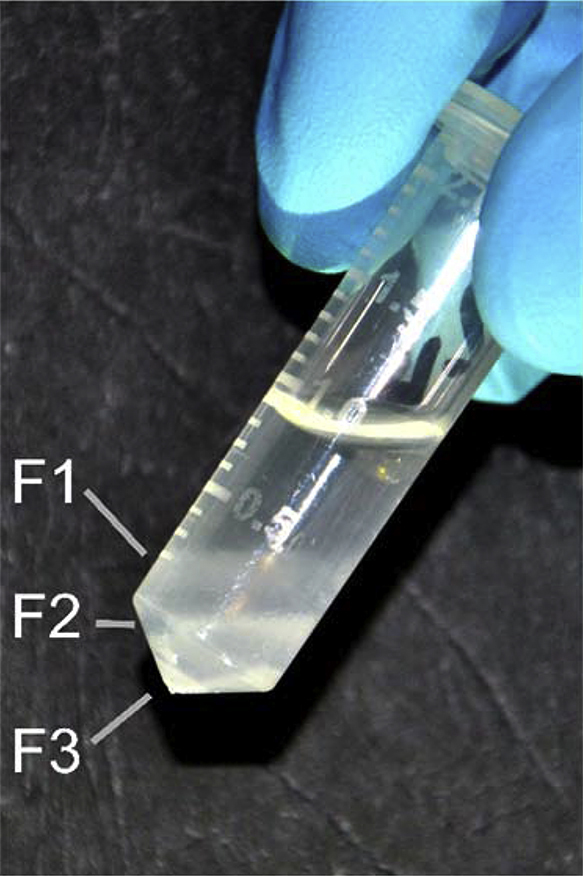
Subfractions of the crude mitochondrial fraction after separation on Percoll gradient. The extrasynaptosomal subfraction (F3) is visible at the tip of the tube, while the synaptosomal one (F2) is found around the inclination of the wall of the tube. The myelin-containing subfraction (F1) is located at the border of the 0% and 15% Percoll layers.

The Percoll gradient containing tubes should be handled with great care by placing into the centrifuge, and spun at 22000rcf (15400 rpm) for 7 min 40 s. In order to save the layers during the centrifugation, the acceleration of the rotor is set to the lowest level and the break is turned off (let the rotor slow down by itself). This way, one can obtain three different layers easily distinguishable by the naked eye: fraction F1—myelin contamination; fraction F2—synaptosomal fraction; fraction F3—perikaryal mitochondria (Fig. 3). After gently removing the tubes from the centrifuge, the layers of interest are recollected and subjected further to cleaning steps. Using a fine pipette, the subfractions of interest can be collected either individually or together depending on the purpose of the experiment. In our research, we are interested in the free and synaptosomal mitochondria, therefore, both the subfraction F2 and F3 are recollected into a clean Eppendorf tube, while the top layer (cell membrane and myelin debris; F1) is discarded.

#### Clearing off the Percoll

At this point, the collected fractions are free of harmful compounds, yet contains a relatively large amount of Percoll that needs to be washed out from our sample. This washing step may differ on the purpose of the experiment. Our goal is to obtain uninjured, coupled, viable mitochondria, so the following centrifugation steps are deployed before the final utilization. The entirely filled tubes of the resuspended sample are spun at 22000rcf (15400 rpm) for 11 min. Since there are no layers anymore, full acceleration and breaking are applied for the fastest centrifugation. After centrifugation, the supernatant can be carefully poured off. As the last step, the remaining, minimal amount of Percoll and EGTA (that might disturb high-precision physiological measurements) is removed. So samples resuspended with 1 ml EGTA free isolation buffer are centrifuged at 13000rcf (11800 rpm) for 11 min, and stored as a pellet in isolation buffer (without EGTA) on 4 °C until the measurements. According to our purpose, in case of hypothalamic samples weighing 18–20 mg, the pellet is resuspended in 50 μl respiration buffer (see later) resulting in around 4.2 mg/ml protein concentration.

### Methods and materials for quality testing

The method is originally developed for mitochondrial respiration measurement purposes, therefore, besides the classical morphological observations, quality testing of the present procedure was mainly focused on viability tests using Clark-type oxygen electrode.

Morphological tests were carried out by electron microscopy: after centrifugation steps, the pellets were immersed into fixative (4% paraformaldehyde, 2% glutaraldehyde) for 24 h, and were further processed as detailed by Zsarnovszky et al. [38].

For viability tests, isolated mitochondria were transferred into respiration buffer (215 mM Mannitol, 75 mM Sucrose, 0.1% BSA, 2 mM MgCl, 2.5 mM KH_2_PO_4,_ 20 mM HEPES [4-(2-hydroxyethyl)-1-piperazineethanesulfonic acid]; pH 7.2) and put into a Clark-type oxygen electrode chamber (Hansatech Instruments, Norfolk, UK) to measure their activity at 37 °C. The electrode groove was filled with potassium chloride for establishing the electrode bridge between cathode and anode. Calibration was fulfilled by air saturated, deionized distilled water in order to establish the air line, while sodium dithionite for zero oxygen line. Using the protocol described by Rosenthal et al. [29], we measured the oxygen consumption by consecutively adding 5 μl pyruvate (500 mM sodium pyruvate in 20 mM HEPES) together with 2.5 μl malate (500 mM malic acid in 20 mM HEPES), 2.5 μl ADP (25 mM in 20 mM HEPES), 1 μl oligomycin (1 mM in ethanol) and 2.5 μl carbonylcyanide-4-(trifluoromethoxy)-phenylhydrazone (1 mM in DMSO [dimethyl sulfoxide]) to the respiration buffer of 500 μl final volume.

The results gained from quality testing were compared to similar protocols, and to the expected mitochondrial function based on the relevant literature e.g. [39], [40].

## Theory behind this method

### Advantages and limitations of Percoll gradient procedures

Considering the characteristics of the different products available for gradient fractionation, and based on our previous experiences, we chose Percoll to obtain high quality and viable mitochondrial fraction for activity measurements. Percoll is a silica sol with non-dialyzable polyvinylpyrrolidone coating, composed by particles of the same diameter (21–22 nm). The principle of fractionation using Percoll is the separation by density rather than size differences: each particle sediments to an equilibrium position where the gradient density is equal to the density of the particle (isopycnic position).

The major advantages of Percoll are the low viscosity (10 ± 5 cP at 20 °C) enabling a rapid procedure, and the very low osmolality (<25 mOsm/kg H_2_O) crucial to avoid shrinkage or swelling of cell organelles [41]. Percoll solutions of different concentration can be easily prepared and layered on top of each other without specialized peristaltic pumps, and the gradient can be stored safely without mixing for hours or even for days (they can be prepared in advance). Important to note that Percoll is considered to be biologically inert, but it is also easy to remove from the sample [19]. The pH of Percoll (9.0 ± 0.5 at 20 °C) can be adjusted in a wide range between 5.5–10 without altering the structure or physical properties of the solution. In addition to these, Percoll suspension possesses physiological ionic strength and pH, it does not penetrate biological membranes, sterilizable and the analysis of gradients is simple with colored density marker beads.

A notable limitation of Percoll procedures is that the distribution of different types of organelles can slightly crisscross among the subfractions, which means in our case, e.g., some synaptosomes might end up in the mainly free mitochondria containing subfraction F3, and *vice versa*. Furthermore, high concentrations of any salts, including HEPES or Tris, can lead to aggregation of membranes and disruption of subcellular fractionation in the gradient [19]. It has to be noted here that these phenomena also emerge in case of other separation media (e.g. Ficoll and sucrose).

### Homogenization

Most of the homogenizing techniques (ultrasonic homogenization, freeze–thawing, hypotonic shock) could easily impair mitochondrial shape and function [31], [42]. For instance, the widely used ultrasonic homogenization, based on the phenomenon of cavitation, causes rapid changes in intracellular pressure producing microscopic air bubbles in the cytoplasm, and damages the mitochondrial membranes [43]. For isolation of viable mitochondria, it is highly recommended to use a motorized (or even a manual) teflon-on-glass Potter-Elvehjem type homogenizer [44]. The clearance and surface shape of the pestle is critical for a sufficient quality (e.g. using grooved grinder is not recommended). In addition, the volume of the glass tube has to be adjusted to the tissue size otherwise a large proportion of the sample would remain on the walls of the tube.

### Making a discontinuous Percoll gradient

Percoll gradient methods refined for brain samples are, in general, for tissue samples weighing more than 1–2 g. These brain blocks, after homogenization (with or without differential fractionation steps), are usually thoroughly resuspended in 2–5 ml isolation solutions and layered on the top of a 10–20 ml gradient column. According to our experiences, hypothalamic samples (weighing 5–20 mg) could be homogenized in 0.5–0.75 ml buffer, at best. Even this small volume needs to be further reduced in order to perform a proper gradient fractionation, otherwise useful fractions cannot be recovered due to the very high gradient solution-fraction ratio [19], [30].

Thus, because of the small size of the targeted brain area, a suitable microcentrifuge tube is needed instead of the commonly used 15–20 ml Corning tubes. The size of the ordinary 1.5 ml Eppendorf tube seemed to be well applicable, however, its tapered shape and beveled lower part ruin the layering and retrieval of the divided fractions. We found that a 2.0 ml microcentrifuge tube with straight sides, conical ending, and with constant wall thickness ensured the gradient formation, and provided the stability of the layers during centrifugation. Depending on the purpose, layering of 0.2–0.5 ml Percoll solutions can be performed manually (using a fine micro-pipette) in the conical microcentrifuge tube, if Percoll content varies in at least 7–10%.

In order to separate somal and synaptosomal mitochondria from other membrane and myelin debris containing fractions, Dunkley et al. introduced a five-layer discontinuous Percoll gradient procedure [19], [45]. Based on their finding, we set up a simplified, two-layer gradient: a 15% Percoll layer on the bottom of the microcentrifuge tube covered by a 0% Percoll containing layer, which is our sample itself. With this gradient, we expect three different layers distinguishable by the naked eye without any specific density marker, if the original tissue block is not smaller than 5 mg (average tissue block that we intended to examine is 10–30 mg). The somal mitochondria sinks to the bottom of the tube, the synaptosomal mitochondria floating in the middle of the 15% Percoll layer but well-demarcable, while the lighter particles, such as membranes and myelin debris, cannot enter the 15% layer, thus it remains on the border of the two layers (Fig. 3). The described two-layer-method focuses on the separation of the mitochondria from other lighter cell organelles and debris, therefore it is advisable to start up from a “crude mitochondrial fraction” that does not contain any larger cellular elements.

Working with Percoll gradients, fixed angle type rotor should be used exclusively since the rotors with swing-out heads can easily disrupt the borders between gradient layers resulting in significantly lower yields.

### Removing Percoll from the fractions

Even though Percoll, according to our present knowledge, is considered as a biologically inert material, after recovering the mitochondrial fractions it must be removed from the suspension. Percoll particles, having relatively high specific surface and space-filling property, can significantly disturb physiological measurements (e.g. by hindering the access of respiration modifiers to the organelles, or it can isolate the measuring electrode surface from the mitochondria). To eliminate the unwanted Percoll, we inserted an additional high speed centrifugation step into our protocol. Note that very high speed and/or long time centrifugation (which could overwhelm Percoll’s impact) might damage the mitochondria [19], therefore, the samples were diluted up to the highest fold, and centrifuged at the lowest possible force that brings the mitochondrial fractions into the pellet as quickly as possible.

## Results and discussion

The presented method yields metabolically active mitochondrial fractions that exhibit good respiratory coupling, high purity, and contain minimal membrane fragment and myelin contamination (that is somewhat higher in the synaptosomal fraction).

**Fig. 4:**
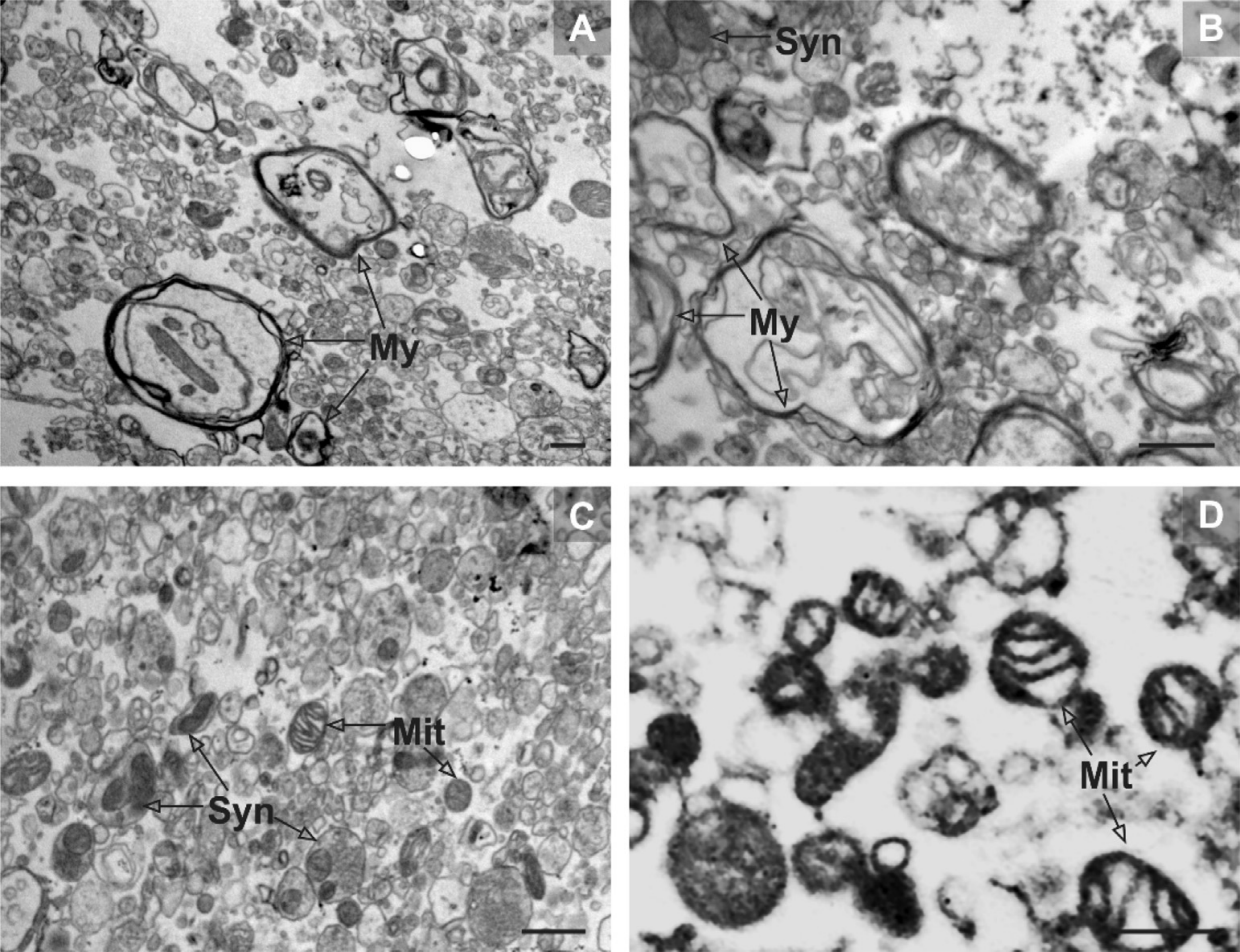
Micrographs of crude and purified mitochondrial fractions. (A) Crude mitochondrial fraction: besides rare mitochondria and synaptosomes, crude mitochondrial fraction is highly “contaminated” by cell debris, membranes and myelin (My). Purification of crude mitochondrial fraction on Percoll gradient enables to gain subfractions enriched in intact mitochondria (fraction F2) and synaptosomes (fraction F3) separated from myelin and cell debris (fraction F1). (B) “Contamination” containing fraction (F1): the cell debris and membranous structures that may destroy mitochondrial assays build the F1 subfraction that is eliminable from the top of the gradient column. (C) Synaptosomal fraction (F2): F2 is predominated by synaptosomes, however contains rare perikaryal mitochondria and small vesicles, as well. (D) Extrasynaptosomal fraction (F3): located in the tip, F3 is highly enriched in well-preserved extrasynaptosomal mitochondria. Moderate mitochondrial density indicates gentle centrifugal forces. Scale bar on each panel represents 1 μm.

**Fig. 5:**
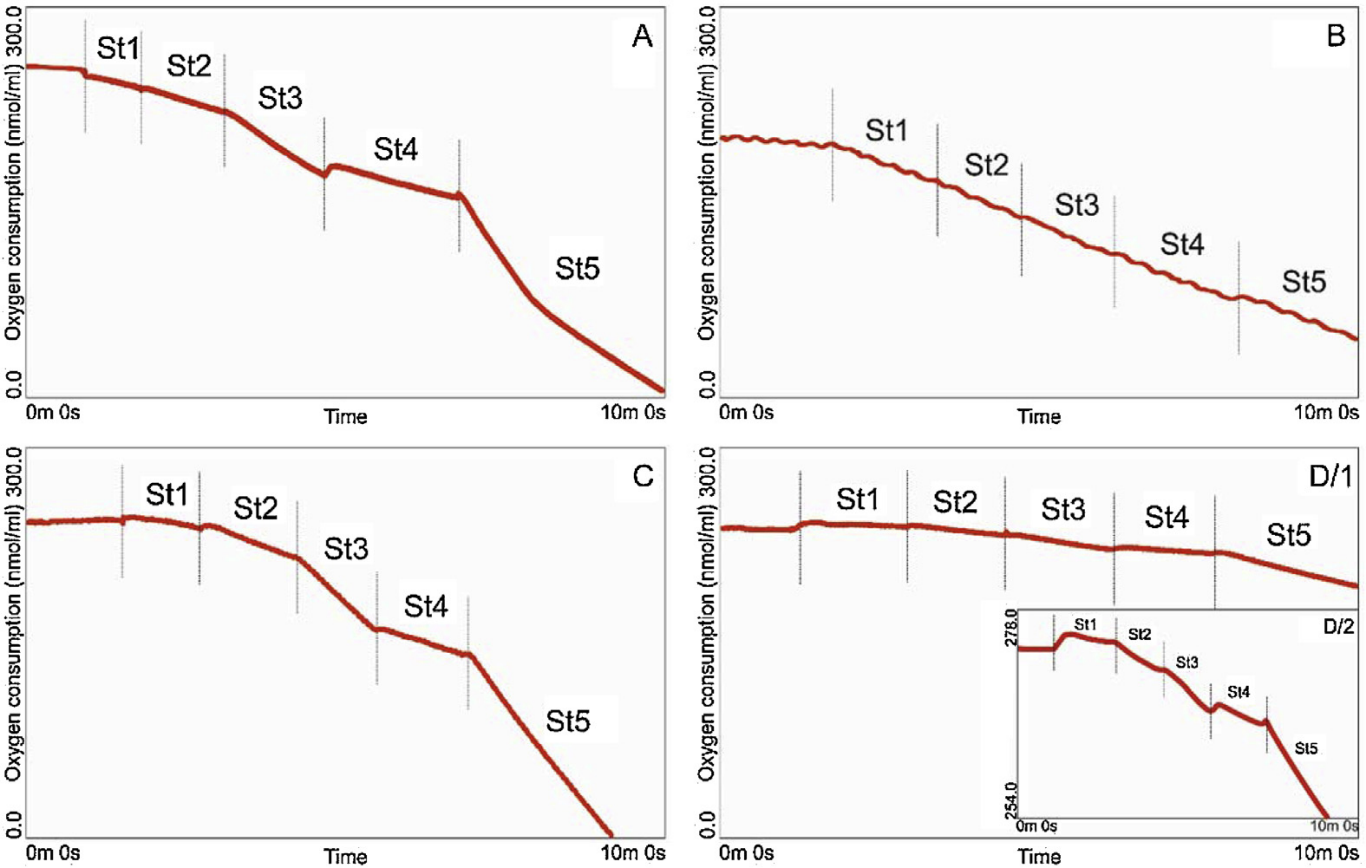
Results of quality testing (mitochondrial respiration measurements). (A) Crude mitochondrial fraction from cortical samples (30 mg): fairly acceptable results gained from cerebral cortex by simple differential centrifugation. (B) Crude mitochondrial fraction from hypothalamic samples (30 mg): same method on hypothalamic samples do not provide physiologically relevant records due to the histological properties of the hypothalamic area. (C) Purified mitochondrial fraction from hypothalamic samples (30 mg): the presented Percoll-based gradient centrifugation method results in a highly responsive, physiologically relevant and viable mitochondrial fraction. (D) Purified mitochondrial fraction from hypothalamic samples (5 mg): the expected responsiveness, and viability can be observed on relatively small hypothalamic samples (5 mg), as well, although the small amount of isolated mitochondria limits the evaluation process (D/1: mrr recording plotted on the same scale as the other curves [A–C]; D/2: to show appropriate responsiveness, Y axis is rescaled to enlarge the same recording).

We tested the viability of our mitochondrial fractions by morphological analysis (electron microscopy, Fig. 4) and measurement of mitochondrial responsiveness in Clark-type oxygen electrode chamber (activity of oxygen consumption, Fig. 5).

### Electron microscopy

In order to check structural integrity and possible disruption caused by the centrifugation steps, particularly those of high speed on Percoll gradient, we sampled the fractions of interest during elaboration of the method to bring them to electron microscopy. Of all possible samples, here we show only those gained from finalized method as representative for quality testing of recovered fractions.

Subfraction F3, located in the tip of the microcentrifuge tube, is highly enriched in well-preserved extrasynaptosomal (free) mitochondria. Its density is moderate indicating gentle centrifugal forces during the fractionating procedure. Subfraction F2 is predominated by mitochondria containing synaptosomes, small vesicles, and larger resealed vesicles of undetermined origin. Membrane structures and intrasynaptosomal mitochondria were intact. Postsynaptic densities attached to synaptosomes can be observed occasionally. Subfraction F1 is dominated by myelin, large vesicles; and rarely, small mitochondria are visible as well as unidentified membranous and granular material (Fig. 4).

### Oxygen consumption measurements

As the most important goal of this protocol was to obtain viable mitochondrial fractions that are suitable for oxygen consumption measurement, at quality testing, we put the major emphasis on the respiratory functions (mitochondrial respiration rate, mrr). Mitochondrial respiration rates were measured and evaluated as described earlier by Toth et al. [34], here we only sum up the general principles of the measurement and the final results of quality testing of the described method. The isolated mitochondria were loaded into a Clark-type electrode chamber, and their oxygen consumption was measured real time (results are expressed as consumed oxygen per minute; nmol O_2_/ml). Five stages (each measured for 60 s) were distinguished according to the subsequently added respiration modifiers [39].

1. State 1 (St1): mitochondrial oxygen consumption in respiration buffer only, without the addition of any substrates that may affect mitochondrial activity. The measured mrr depends on the actual metabolic state of the mitochondria.
2. State 2 (St2): mitochondrial function in the presence of oxidative substrates (pyruvate and malate in a final concentration of 5 mM and 2.5 mM, respectively) of the Krebs’ cycle, but in lack of added substrate for the ATP synthase [16]. Under such conditions, Krebs’ cycle intensifies and oxygen consumption increases due to consequential facilitation of the terminal oxidation and oxidative phosphorylation if the down-regulating mechanisms are not active. Mitochondrial respiratory rate measured in St2 is limited by the amount of endogenous ADP present in the mitochondria.
3. State 3 (St3): state 3 is initiated by adding ADP in a final concentration of 130 μM. Being the substrate for ATP synthase, ADP is a major upregulator of mitochondrial respiration. Under such conditions, mrr increases if prior fuel supply of the hypothalamic tissue was sufficient. Therefore, if excess amount of ADP is added to the sample (Krebs’ cycle is already fueled up), oxidative phosphorylation is limited exclusively by the activity of ATP synthase.
4. State 4 (St4): in state 4, oligomycin (2.5 μM in the final concentration) is used to block the ATP synthase activity, therefore the oxidative phosphorylation, however the steps of terminal oxidation continues [46]. Under such conditions, oxygen consumption depends on the actual uncoupled stage and the activity of alternative oxidases of the mitochondria. Under physiological conditions, uncoupling and alternative oxidation play important roles in transient down-regulation of ATP biosynthesis when cellular energy needs drop. In case of fully viable mitochondria, oligomycin results in remarkably reduced mrr compared to that observed in state 3. It is to note that improper purification of the sample may lead easily to elevated state 4 mrr rendering a reliable evaluation unable.
5. State 5 (St5): at last, FCCP [carbonylcyanide-4-(trifluoromethoxy)-phenylhydrazone] is added to the sample in a final concentration of 5 μM. FCCP is a cyanide derivative that functions as protonophore dissipating mitochondrial membrane potential by artificially carrying protons across the inner membrane, thus causing uncoupled respiration [47], [48]. Decreased oxygen level under such conditions depends on the initial (*in vivo*) metabolic state of the sampled tissue, and the amount of oxygen consumed during states 1–4 respiration. Therefore, this experimental setup is also known as total mitochondrial respiratory capacity.

By the application of the above description, one should take into consideration that most studies available on mitochondrial respiratory functions examine samples larger than that of our interest, or grouped samples of smaller brain areas obtained from several animals [49], [50]. Thus, we compered our results to observations gained from respiratory measurements on cortical and hypothalamic samples of bigger size (other species and/or grouped samples) [30], [51], [52].

Fig. 5 intends to demonstrate the benefits and limitations of the presented. It is important to note that the main goal of the figure is not to provide a direct comparison, but to highlight advantages of the presented protocol. Before the Ficoll and Percoll era, brain samples for mitochondrial measurements were purified mainly using exclusively differential fractionation (that yields merely crude mitochondrial fraction) [9]. Interestingly, applying to cortical samples, this method provides quite well-evaluable records almost as good in quality as those obtained from gradient fractionation (Fig. 5A). Therefore, these widely simplified and quick procedures can provide approximately reliable data in case of cortical (or cerebellar) samples [31]. Most probably, this is due to the relatively homogenous tissue organization of the cortex, where the ratio of the microstructural elements that can disturb mitochondrial bioenergetics is very low. This allows us to use the crude mitochondrial fraction as an external control of our method (Fig. 5A). On the demonstrated example, one could observe the appropriate responsiveness of the sample to the respiration modifiers in all five mrr stages (described above). The same procedure on hypothalamic samples (or possibly on other samples of high tissue heterogeneity) never results in similarly appropriate records (on the mrr recording: a slope of continuous and unbroken inclination, where no respiratory modifiers could alter the oxygen consumption; Fig. 5B). This might be due to the above-mentioned uncoupling effect of lipid-like contaminants deriving from the white matter and the extracellular matrix [36], [37]. Acting as uncouplers, contaminating molecular structures provoke leakage of the inner membrane leading to an abnormally high oxygen consumption rate in all experimental mitochondrial respiratory states during registration [53]. Thus, the consumption rates recorded from such brain areas are attributed, in an unknown degree, to the leakage that hinders the analyses of the native mitochondrial activity itself.

Phases of mitochondrial respiratory measurements: St1: mitochondrial sample in respiration buffer only; St2: pyruvate and malate added to fuel Krebs’ cycle; St3: ADP added to measure ATP synthase activity; St4: ATP synthase blocked by oligomycin to observe alternative oxidation and native uncoupling; St5: uncoupler FCCP added to estimate mitochondrial readiness.

Based on previously published descriptions [48], [54] and on our experience derived from the comparative analyses of mrr recordings [34], [55], we can state that the presented Percoll-method ensures the separated extraction of physiologically viable (not leaking and well-coupled) mitochondrial fractions (Fig. 5C) from at least 5 mg hypothalamic block, and it enables reliable respiratory recordings (Fig. 5D). Major evidences are the shape of the mrr curve in all stages that is comparable with the literature data [29], [39], [40], and the acceptable responsiveness of the sample in each respiration stages (Fig. 5D/2).

### Limitation of the presented method

The main limitation of the presented method comes from the size of the isolated tissue block. Workable size of the original sample depends on the volume of isolation buffer: what is the smallest volume that the researcher and the available instruments can reliably work with. For our experimental purposes, the Hansatech instrument fits the most, therefore this method was adjusted to this instrument. Certainly, the presented procedure should be obviously readjusted to available devices. The lowest chamber volume, in which the mitochondrial measurement can be carried out, is practically about 250 μl. We have tested several tissue blocks of different sizes between 1 and 35 mg, and due to the dilution of the fractionated mitochondrial sample in the 250 μl chamber volume, the smallest size of the original tissue block, from which the mrr recording remained still evaluable, resulted to be not less than 5 mg (Fig. 5D). Respiratory rates of lower sample size did not exceed the background error of the instrument.

To our present knowledge, this method provides the easiest and shortest procedure that could gain fully purified and viable mitochondria extracted from a brain tissue of small size. There are similar methods available that could be applied for brain tissues not smaller than 40–80 mg, but those omit purification steps essential for mitochondrial oxygen consumption measurements, moreover they are not tested on hypothalamic samples [21], [31], [56].

## Conclusions

In the article, we have demonstrated a fully detailed protocol that enables gaining viable mitochondria from brain areas with heterogeneous histological microstructure (e.g. hypothalamus) within 3 h. One of the important advantages of the method is that the original sample size can be reduced even to 5 mg (or even smaller depending on brain region and available equipment). As the result of this method, mitochondrial subfractions are visible with naked eye and easy to separate for further high-precision examinations, such as biochemical or mitochondrial respiration measurements. This way, we have a tool in our hand, through which we might be able to examine mitochondria of small, isolated brain parts; and by combining this method with mitochondrial respiration measurements, we have an outstanding opportunity to get new insights into the neuronal energetics.
