# Supplementary material for: Preparation of purified perikaryal and synaptosomal mitochondrial fractions from relatively small hypothalamic brain samples
Source: MethodsX. 2016 May 19;3:417–29. doi: 10.1016/j.mex.2016.05.004 (PMC4887559; doi:10.1016/j.mex.2016.05.004)
Supplement: Supplementary file 1 [file mmc1.docx]

## Appendix A

## Detailed protocol

1. **Preparation of Percoll gradients**
   1. ***Making and diluting a stock solution of Percoll***
2. Dilute Percoll adding 9 parts (v/v) of Percoll to 1 part (v/v) of 2.5M sucrose solution.
3. Filter Percoll through a Millipore AP15 prefilter.

Aggregated particles can lead to disruption of normal subcellular fractionation.

Filtered and aliquoted Percoll solution can be stored at 4 °C for few days, or for longer time at -20 °C, however, it has to be refiltered before use.

1. Adjust the pH to 7.2 with 37% HCl and 3M KOH solutions.
2. Dilute the Percoll stock solution with isolation buffer to get the required 15% gradient solution.
   1. ***Preparation of a discontinuous Percoll gradient***
3. Fill a 2ml conical Eppendorf tube with 500µl of 15% Percoll solution.
4. Store this on ice until “crude mitochondrial fraction” is ready (that will constitute the 0% Percoll layer).
5. **Preparation before animal sacrifice**
6. Prepare:
   1. For one sample, label 3 normal Eppendorf tube and one 2ml conical tube filled with 500µl of 15% Percoll solution.
   2. Cool down the surgical tools (brain matrices, scissors, scalpels, blades, forceps, guillotine, Petri-dish with nylon mesh on it), the teflon-on-glass homogenizer, isolation buffer with EGTA.

It is of utmost importance that all of the tools used to dissect the brain (brain matrix, blades, scissors, spatulas, etc.) must be perchilled and kept on ice at 4°C.

1. **Dissection and tissue homogenization**
2. For each tissue sample, fill one labeled normal 1.5ml Eppendorf tube with 750μl, ice-cold isolation buffer .

The isolation buffer should not be older than a week, otherwise the pH could shift into unfavorable range.

The volume of isolation buffer cannot exceed 750μl, because, after the first centrifugation step, the pellet will be resuspended in 750μl isolation buffer again, recentrifuged and finally the two sets of supernatants (both around 750μl) will be poured together to top the 1.5ml Eppendorf tube.

1. Extract the needed brain region from the sacrificed animal (in our case the hypothalamus). The dissection of the brain must be done in ice cold environment.
   1. Decapitate the animal, remove the skin and muscles from parietal and occipital regions of the skull.
   2. Make a horizontal incision of the skull on both sides from the *foramen magnum* in the direction of the ears (*external auditory canal*). Remove the basal region ventrally from the incision. Make a cut along the *satura lambdoidea* from the ears up to the top of the skull on both sides. Remove the parietal region of the skull. Make a single cut along the sagittal suture through the *parietal* and *frontal* bones. Open up the skull by turning out the two halves of the parietal-frontal bones.
   3. Lift up the brain slightly by the cranial end with the cold spatula, cut through the optic nerve with the finest pair of scissors. Lift up and remove the brain from the skull, and place it on the Petri-dish or brain matrix being on ice.
   4. Remove the connecting tissue from the basal part of hypothalamus with a fine forceps.
   5. Make vertical incisions using the brain matrix and ice cold blades.

*For a coronal section of the entire hypothalamus:* Make an incision right behind the caudal part of the *chiasma opticum* (Bregma -0.25), and an other one through the *corpus mamillare* (Bregma -5.0). Lay the gained coronal section with the cranial side down on the Petri-dish, then first, remove the *piriform* and *entorhinal cortex*, then the thalamic area dorsal of the *fornices*. Make sure that you removed all the possible white matter parts in the border region between the hypothalamus and the adjacent cortex, and the trunks of the *chiasma opticum* (they can be harmful at the mitochondrial measurement as mentioned above).

Without a brain matrix, you can also fix the brain with your fingers by the cerebellum and brain stem, then the afore-mentioned vertical incisions can be easily carried out.

According to other purposes, the hypothalamus can be further divided either with coronal cuts using the brain matrix and/or, having a particular coronal section lying on the Petri-dish, with help of micro scalpels and syringe needles (punch method) to separate longitudinal subregions.

- 1. Having your tissue block, the binding tissues, blood vessels and the meninges must be removed from peripheral parts of the tissue block as carefully as possible, but relatively quickly (in less than 10 seconds).

1. Put the tissue block (5-60mg) as quickly as possible into 750μl ice-cold isolation buffer. Store the tubes this way for a minimum period of time while all the required samples of one or more animals are prepared similarly.
2. Transfer the content of the microcentrifuge tubes one-by-one into the teflon-on-glass tissue homogenizer and homogenize the blocks with ten strokes (moving the glass tube firmly up and down) using a motorized pestle set on 600-800rpm.

The tissue grinder tube and pestle should be perchilled and kept on ice during homogenization. This can be achieved holding the grinder tube, with the pestle in it, in a beaker filled with ice-cold water before and during the homogenization.

1. After the homogenization, collect as much buffer and foam as you can, and put it into a 1.5ml Eppendorf tube. The resulting homogenate could be kept on ice while other tissue samples are prepared.
2. Clean the homogenizer between to samples with isolation buffer.
3. **Preparing crude mitochondrial fraction from brain tissue**
4. Spin all the tubes for 4 minutes at 1300rcf (3700rpm), 4°C.
5. Save supernatant into empty tube, place it on ice.
6. Resuspend pellet in 750µl isolation buffer with EGTA.
7. Spin resuspended pellet at 1300rcf (3700rpm) for 4 minutes, 4°C.
8. Collect supernatants from step 3 and step 5 into one tube (at this point, the tube with pellet can be discarded).
9. Spin the two supernatants at 13000rcf (11800rpm) for 11 minutes, 4°C.
10. Discard the supernatant by aggressively flicking it off (it contains no more useful elements).
11. Resuspend the pellet with 500µl of isolation buffer with EGTA, which will be transferred onto the 15% Percoll (already prepared earlier).
12. **Percoll gradient fractionation procedure**
13. Put the resuspended pellet from step 4.8 on the top of the prepared 15% Percoll layer (see above) in the 2ml conical tube. In this, avoid even the slightest mixing of the two layers.
    1. To do so, first move the tip of the pipette slowly along the wall of the microcentrifuge tube and touch extreme gently the surface of the Percoll layer.
    2. Then pull the tip back up making a narrow fluid bridge between the tip and the existing surface. This bridge will lead the suspension of sample to flow gently to sit on the surface of the 15% Percoll layer.
    3. Next, start releasing the sample very slowly. Ensure that the pipetted fluid will not enter into the 15% Percoll layer, but will expand on its surface to build the upper layer.
    4. Pipette the full amount of suspension (i.e. 0% Percoll layer containing the sample). Avoiding bubbles that can disturb the sedimentation of the organelles during the high speed centrifugation.
    5. If well done, you will see a clean, straight line between the layers, visible only when holding the tube in the direction of light, and fades after circa 30 minutes.
    6. Put the gradient containing tubes into the centrifuge as gently as possible.
14. Centrifuge the gradients at 22000rcf (15400rpm) exactly for 7 minutes 40 seconds (it results 7 minutes on top speed), 4°C. Set both the acceleration and brake force on the minimum rate available.

Sudden speed-up and stop can significantly disturb the layers on the gradient, rendering them blurry.

1. Remove the Percoll tubes from the rotor very carefully, and put them back on ice.
2. Three whitish layers are visible with naked eye. The uppermost contains the cell membrane and myelin debris, the middle contains the synaptosomes, and finally the lowest holds the somal mitochondria.
3. The two fractions of interest can be collected individually or together depending on the purpose of the experiment.
4. Lead the tip of pipette slowly down to the required layer going through the uppermost myelin rich region without suck any of it.
5. For both mitochondria containing layers, collect the solution from the bottom of the tube very carefully with a medium (200µl) pipette.
6. **Removal of Percoll from the sample**
7. After the Percoll procedure, put the samples into a new Eppendorf tube, and resuspend it with isolation buffer containing EGTA, finally, top off the tube.
8. Spin at 22000rcf (15400rpm) for 11 minutes (at 4°C; full brake and full acceleration can be used).
9. Carefully pour off the supernatant as the pellet is still loose due to its Percoll content. Intense sucking or pouring can easily result in losing the important fractions.
10. Resuspend the pellet with buffer with 1ml isolation buffer (without EGTA)**.**

Presence of EGTA, applied for buffering calcium ions in physiological solutions, can disturb the mitochondrial respiration measurement as well as many protein assays, biochemical measurements, etc.

1. Centrifuge at 13000rcf (11800rpm) for 11 minutes (at 4°C).
2. The samples can be stored together with the supernatant till measurements.
3. For respiration measurements, pour off the supernatant and resuspend the pellet 50µl of respiration buffer right before the beginning of the measurement.
