# Supplementary material for: Preparation of purified perikaryal and synaptosomal mitochondrial fractions from relatively small hypothalamic brain samples
Source: MethodsX. 2016 May 19;3:417–29. doi: 10.1016/j.mex.2016.05.004 (PMC4887559; doi:10.1016/j.mex.2016.05.004)
Supplement: Supplementary file 2 [file mmc2.docx]

## Appendix B

## Reagents and equipment

1. **Reagents for preparation of buffers and for the fractionation**

BSA bovine serum albumin; fatty acid free (!); Sigma, cat. no. A7511

EGTA ethylene glycol-bis (2-aminoethylether)-N,N,N’,N’-tetraacetic acid, C_14_H_24_N_2_O_10_; RNA, DNA free (!); Sigma, cat. no. E3889

HEPES HEPES potassium salt, 4-(2-Hydroxyethyl)piperazine-1-ethanesulfonic acidpotassium salt, C_8_H_17_KN_2_O_4_S; Sigma, cat. no. H0527

HCl hydrochloric acid 37%; Carlo Erba, cat. no. 403871

KH_2_PO_4_ potassium phosphate monobasic, H_2_KO_4_P; Sigma, cat. no. P5655

KOH potassium hydroxide; Sigma, cat. no. P1767

Mannitol D-Mannitol, C_6_H_14_O_6_; Sigma, cat. no. M9546

MgCl_2_ magnesium chloride solution; Sigma, cat. no. M1028

Sucrose α-D-glucopyranosyl β-D-fructofuranoside; C_12_H_22_O_11_; Sigma, cat. no. S7903

Percoll GE Healthcare, cat. no. 17-0891-01

1. **Buffer solutions**

To set pH 7.2 use the 5M KOH and the 37% HCl solutions. These buffers should be stored in a reagent bottle at 4°C for not more than a couple days.

| **Isolation buffer with EGTA** | **Isolation buffer without EGTA** | **Respiration buffer** |
| --- | --- | --- |
| 215mM Mannitol  75mM Sucrose  0.1% BSA  1mM EGTA  20mM HEPES | 215mM Mannitol  75mM Sucrose  0.1% BSA  20mM HEPES | 215mM Mannitol  75mM Sucrose  0.1% BSA  2mM MgCl  2.5mM KH_2_PO_4_  20mM HEPES |

1. **Equipment**

- Filtration apparatus for preparation of Percoll solution applied with 90mm diameter Millipore AP15 prefilter (glass fiber filter; Millipore, Billerica, MA, USA; cat. no. AP1509000)
- pH meter (e.g. SevenEasy S20; Mettler Toledo; Schwerzenbach, Switzerland)
- Rodent guillotine (DCAP; Kent Scientific, Kent, UK)
- Nylon mesh (pore size 0.45μm, diam. 90mm; Sigma, cat. no. Z290785)
- Brain matrix (rat 175-300g, 0.5mm coronal, stainless steel; World Precision Instruments, Sarasota, FL, USA; cat. no. RBMS-300C)
- 10-15ml teflon-glass tissue grinder of type Potter-Elvehjem applied with a motor driven (capable of 500 and 800rpm.) pestle of 0.1–0.15mm clearance. (e.g. Wheaton (Millville, NJ, USA; distributed by Thermo Fisher Scientific, Waltham, MA, USA)

Note that all the properties of the grinder (volume of its tube, clearance, number of strokes, speed of pestle rotation) must be suitable for the eventual size of the tissue block undertake the homogenization procedure.

- 1.5ml transparent microcentrifuge tubes (Eppendorf tubes, natural; VWR International, Radnor, PA, USA; cat. no. 700-5239, 3810X)
- 2.0ml transparent conical microcentrifuge tubes (Eppendorf tubes, natural; Deltalab, Barcelona, Spain; cat. no. 4092.6N)
- Bench-top centrifuge capable of running a Beckman J2M1, J2-21, JA-20, Avanti J-26XPI or similar fixed angle rotor. We used a Hettich Universal 32 centrifuge (Hettich Instruments, Beverly, MA, USA) applied with a rotor no. 1689 (30 sleeve, fixed 45° rotor.

Note that the timing of centrifugation steps in this protocol includes the acceleration time based on the depth and angle of this rotor.
